# Structural Features of 1,3,4-Thiadiazole-Derived Ligands and Their Zn(II) and Cu(II) Complexes Which Demonstrate Synergistic Antibacterial Effects with Kanamycin

**DOI:** 10.3390/ijms21165735

**Published:** 2020-08-10

**Authors:** Dariusz Karcz, Arkadiusz Matwijczuk, Daniel Kamiński, Bernadette Creaven, Ewa Ciszkowicz, Katarzyna Lecka-Szlachta, Karolina Starzak

**Affiliations:** 1 Department of Analytical Chemistry (C1), Faculty of Chemical Engineering and Technology, Cracow University of Technology, 31155 Kraków, Poland; karolina.starzak@pk.edu.pl; 2 Department of Biophysics, University of Life Sciences in Lublin, 20-950 Lublin, Poland; arkadiusz.matwijczuk@up.lublin.pl; 3 Department of General and Coordination Chemistry and Crystallography, Institute of Chemical Sciences, Maria Curie-Sklodowska University in Lublin, 20-031 Lublin, Poland; dkami@umcs.pl; 4 School of Chemical and Pharmaceutical Sciences, Technological University Dublin, Kevin St., D2 Dublin, Ireland; bernie.creaven@tudublin.ie; 5 Department of Biotechnology and Bioinformatics, Faculty of Chemistry, Rzeszow University of Technology, 35-959 Rzeszów, Poland; eciszkow@prz.edu.pl (E.C.); szlachta@prz.edu.pl (K.L.-S.)

**Keywords:** 1,3,4-thiadiazole, thiadiazole ligands, thiadiazole complex, antibacterial, antioxidant, neurodegeneration, synergistic effect, kanamycin

## Abstract

Classical synthetic protocols were applied for the isolation of three novel 1,3,4-thiadiazole derivatives which were then complexed with the biologically important Cu(II) and Zn(II) ions. All free ligands and their corresponding complexes were characterized using a number of spectroscopic techniques including Ultraviolet-visible (UV–vis), Fluorescence, Infrared (FT-IR), tandem liquid chromatography-mass (LC-MS), X-ray diffraction (XRD), and Nuclear Magnetic Resonance (NMR) spectroscopy (^1^H, ^13^C, HSQC, HMBC). The results obtained are consistent with the formation of dihydrate complexes, in which the chelation of the metal ion occurs via one of the thiadiazole nitrogen atoms and the deprotonated hydroxyl group of the neighboring resorcynyl moiety. The Zn(II) complexes utilize a 1:1 ligand–metal ratio, while in the Cu(II) complexes the ligand–metal ratio is 2:1. Although the antibacterial testing identified moderate activity of the compounds against the tested bacterial strains and additionally modest antioxidant activity, a strong synergistic antibacterial effect against *Staphylococcus aureus*, using concomitant treatment of thiadiazole derivatives with the commercial antibiotic kanamycin, was observed. The most active thiadiazole derivative demonstrated a minimal inhibitory concentration (MIC) of 500 μg/mL while it was 125 μg/mL in the presence of kanamycin. Moreover, in the presence of few thiadiazole derivatives the MIC value of kanamycin decreased from 0.39 μg/mL to 0.5 μg/mL. The antioxidant activity (IC_50_) of the most active thiadiazole derivative was determined as 0.13 mM which was nearly three-fold lower compared to that of TROLOX (0.5 mM).

## 1. Introduction

Thiadiazoles are an important group of five-membered heterocycles demonstrating extraordinary physiochemical properties including dual fluorescence emission [1,2,3], crystal solvatomorphism [4], and keto-enol-like tautomerism [5]. Numerous reports have highlighted the antimicrobial, anticancer, antioxidative, or anticonvulsant activities as characteristic of 1,3,4-thiadiazoles [6,7,8]. Due to these interesting features the thiadiazole-derived compounds were extensively studied in our group [9,10,11,12,13], with several 1,3,4-thiadiazole derivatives reported to possess significant acetylcholinesterase (AChE) and butyrylcholinesterase (BuChE) inhibition activities rendering them as potential anti-neurodegenerative agents [14].

Inhibition of AChE and BuChE enzymes is one of the existing approaches taken in the design of novel anti-neurodegenerative agents. Given that neurodegenerative disorders may result from a perturbed homeostasis of essential metals such as Cu(II) and Zn(II) [15,16], engineering of novel metal chelators possessing AChE and BuChE inhibitory ability has been proposed as a new approach to the treatment of neurodegenerative disorders such as Alzheimer’s or Parkinson’s diseases [17,18,19,20].

Our previous studies on thiadiazole derivatives have focused on examination of the metal-binding ability of 1,3,4-thiadiazoles bearing the *o*-hydroxyphenyl moiety at C5 carbon, which were used for the isolation of a series of Zn(II), Cu(II), and Pd(II) complexes [21]. The spectroscopic characterization of these complexes revealed significant structural differences in their metal–ligand ratios depending on the central metal type; however, in all those complexes the *o*-hydroxyphenyl moiety together with the neighboring thiadiazole nitrogen were identified as the metal binding sites. These findings were consistent with widely reported fact that thiadiazole-derived ligands may demonstrate versatile coordination modes which strongly depend on the presence of additional substituents [8,22,23,24]. This diversity is particularly high in case of the coordination to Cu(II) and Zn(II) ions, which are well-known for their ability to adopt a wide variety of coordination modes [25,26].

**Figure 1:**
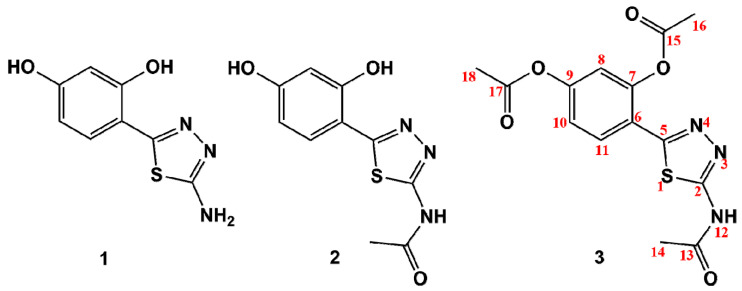
Structures of the 1,3,4-thiadiazole-derived ligands **1**, **2**, **3**, and the numbering system of atoms (red) used for the assignment of the ^1^H and ^13^C NMR chemical shifts.

Our current studies focus on the isolation of 1,3,4-thiadiazole ligands **1**–**3**, which would keep their metal-chelating ability, while offering the possibility for additional structural modifications (Figure 1). Therefore in this work, the *o*-hydroxyphenyl attached to the C5 carbon of the thiadiazole ring remained a main structural motif, while the C2 position was substituted by the simple -NH_2_ group, as the family of 2-amino-1,3,4-thiadiazoles belong to the most extensively studied thiadiazole derivatives. Secondly, regardless of the fact that the most biologically active 2-amino-1,3,4-thiadiazoles are usually substituted with an aromatic ring at their C5 carbon, such tandems with polyphenolic moieties are limited. This relative scarcity prompted us to revisit the classical synthetic route in the 1,3,4-thiadiazloes synthesis aiming at obtaining 2-amino-2-(2,4-dihydroxy)-1,3,4-thiadiazole **1** as a model ligand for subsequent reaction with Zn(II) and Cu(II) salts. Introduction of additional reactive substituents, and especially the lone electron pair donors such as -OH and -NH_2_, is associated with an increase in the number of potential metal-binding sites. In order to assess the possibility for alternative coordination modes, compound **1** was modified by acetylating its -NH_2_ and both its -OH groups. The metal coordination ability of the resulting mono-acetylated and tri-acetylated derivatives (**2** and **3**, respectively) was compared to that of **1** (Figure 1). The structures of all compounds were elucidated using spectroscopic methods. Moreover, antioxidant activity testing and antimicrobial screening against a limited number of bacterial strains was performed on the thiadiazole free ligands and their Zn(II) complexes. Therefore, the main aim of our current work was an isolation and structural elucidation of newly synthesized thiadiazole derivatives and spectroscopic examination of their metal-binding ability. Secondly, given the fact that the thiadiazole derivatives and especially the metal complexes obtained are novel, their antibacterial and antioxidant activity was assessed for a first time. Thirdly, based on the synergistic antifungal effects that are characteristic of the structurally similar thiadiazoles [12], a possibility for the synergism with commercial antibacterial agent, kanamycin, was examined. Our studies were driven by hypotheses that determination of the metal-binding ability of thiadiazole ligands obtained may be a new approach to treatment of neurodegenerative disorders, while the assessment of their synergistic interactions with known antibiotics may shed new light on the formulation of more effective antibacterial medicines.

## 2. Results

### 2.1. Synthesis of 1,3,4-Thiadiazole-Derived Ligands and Their Cu(II) and Zn(II) Complexes

**Figure 2:**
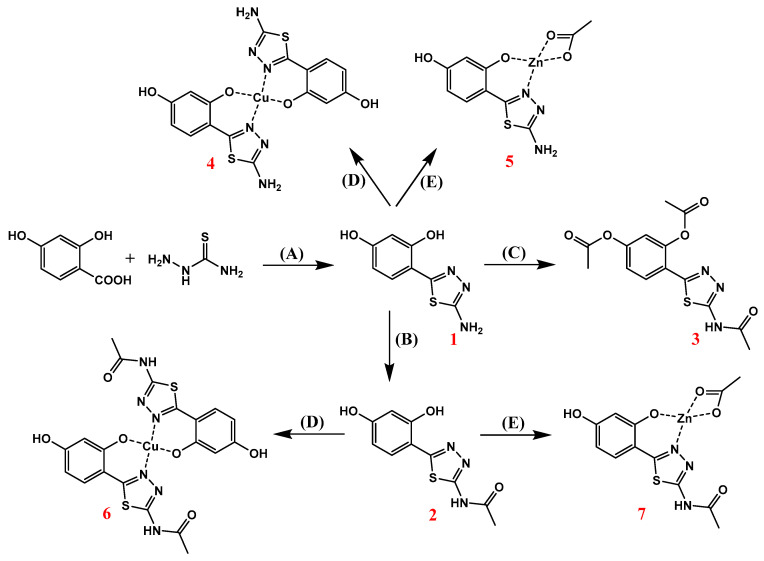
Synthetic pathway for the synthesis of 1,3,4-thiadiazole-derived ligands **1**–**3**, and their corresponding Cu(II) and Zn(II) complexes **4**–**7**: (**A**) POCl_3_, 75 °C; (**B**) Ac_2_O, H_2_O, reflux; (**C**) Ac_2_O, H_2_SO_4_, reflux; (**D**) Cu(CH_3_COO)_2_xH_2_O, MeOH/H_2_O, reflux; (**E**) Zn(CH_3_COO)_2_xH2O, MeOH/H_2_O, reflux. For better clarity, the hydrated water was omitted in the structures of complexes.

In the first synthetic step 2-amino-5(2,4-dihydroxyphenyl)-1,3,4-thiadiazole **1** was obtained as result of the classical POCl_3_-mediated reaction between 2,4-dihydroxybenzoic acid and thiosemicarbazide. Compound **1** was then acetylated with use of aqueous acetic anhydride yielding the amide derivative **2**, while the addition of catalytic amount of H_2_SO_4_ allowed the formation of fully acetylated derivative **3**. All thiadiazole derivatives **1**–**3** were then reacted with Cu(II) and Zn(II) acetate salts, which in the case of compounds 1 and 2 resulted in the formation of their respective Cu(II) and Zn(II) complexes **4**–**7**. Compound **3** did not form any metal complexes with the metal salts used. The synthetic pathway illustrating the syntheses of thiadiazole derivatives is given in Figure 2.

### 2.2. H-NMR Spectroscopy

The ^1^H-NMR spectrum of **1** consists of six signals originating from the resorcynyl ring and the -NH_2_ group. The two most downfield-positioned broad signals at 10.91 and 9.84 ppm result from the phenolic -OH groups, while the -NH_2_ substituent gives rise to a broad singlet at 7.15 ppm. These signals come from exchangeable hydrogen atoms and disappear upon the treatment of sample with D_2_O (the D_2_O exchange spectrum not shown). The remaining three signals present in the aromatic region are assigned based on their multiplicities, coupling constants, and integration (Table 1, Figure S1). In more detail, the double doublet at 6.36 ppm represents the H10 proton coupling with another two doublets at 7.53 and 6.38 ppm, which originate from the respective H11 and H8 protons.

Selective N-acetylating of **1** with subsequent formation of **2**, manifests in the disappearance of the amine peak and gives rise to two new signals, namely the highly deshielded amide singlet at 12.32 ppm and an aliphatic -CH_3_ singlet at 2.18 ppm. Moreover, a significant sharpening of both -OH signals as well as a slight downfield shift of the aromatic peaks is observed (Table 1, Figure S2).

Simultaneous acetylating of the amine and both phenolic groups in **1** resulted in the formation of **3**, with the subsequent replacement of their respective signals with a series of singlets characteristic of two methyl esters and one amide moiety formation (Table 1). Also, compared to those of **1** and **2**, further downfield shifts of the remaining signals were observed (Figure S3).

**Figure 3:**
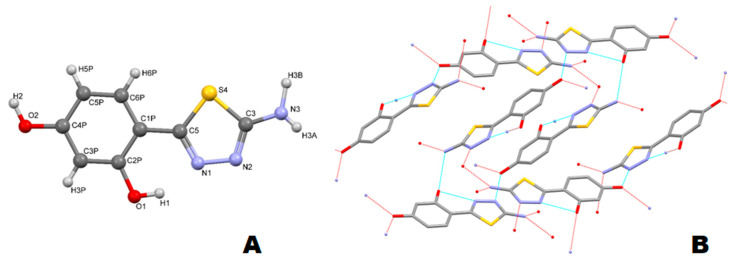
Crystal structure of thiadiazole **1**. (**A**) Atoms are numbered according to the cif file (see supplementary material); (**B**) Packing of the molecules in a crystal net (for better clarity, the hydrogen atoms are omitted).

The ^1^H-NMR spectra of thiadiazole derivatives **1**–**3** revealed the presence of additional set of low intensity signals identified as originating from a series of isomers, which may form due to a possibility for the rotation around the thiadiazole-resorcynyl-linking bond. Based on the integration of two sets of signals it has been determined that the ratio between the two main isomers does not exceed 1:10 and is most likely solvent-dependent. Although the presence of isomers makes the ^1^H-NMR spectra slightly more complicated, neither HPLC-MS nor microanalysis data indicated the presence of significant amount of other impurities. Therefore, the structure of the major isomer was assumed as identical to that obtained from X-ray diffraction analysis (Figure 3A).

Due to their sparing solubility, the Zn(II) complexes **5** and **7** did not give clear NMR spectra. Nevertheless, in both cases the signal at approximately 1.8 ppm was present, evidencing the key structural feature of Zn(II) complexes formed, namely the acetate ion partaking in coordination around the metal center. These results were consistent with microanalysis data, which correlated best with the structure consisting of one thiadiazole-derived ligand and one acetate ion coordinated to the Zn(II) central metal. Moreover, the residual water peak was unusually broadened which may be an evidence for the presence of additional aqua ligands. The NMR spectra of Cu(II) complexes **4** and **6** were not acquired due to their paramagnetic character, which effects in extended T2 relaxation times, thus significant broadening of NMR signals.

### 2.3. C-NMR Spectroscopy

The ^13^C-NMR signals in thiadiazole ligands were assigned based on a routine ^13^C NMR spectrum and additional 2D-NMR experiments (HSQC, HMBC). As expected, compound **1** shows 8 carbon signals. Compared to the ^13^C NMR spectrum of **1** the spectrum of **2** shows an additional two carbon signals due to the amide moiety, while the fully acetylated compound **3** has six additional peaks (Table 2) (Figures S4–S6).

### 2.4. X-ray Diffraction

The asymmetric part of unit cell contains one planar molecule. In the solid state the rotation in the molecule is prevented by a hydrogen bond between the phenolic *o*-hydroxyl group and the neighboring N atom of the thiadiazole ring. Both hydroxyl groups are involved in the formation of intermolecular hydrogen bonds (Figure 3B). The *o*-hydroxyl group forms an intramolecular H-bond with the amine moiety of neighboring molecule with a distance A..H of 2.236 Å. The second phenolic -OH interacts with two neighboring molecules acting as both an acceptor of hydrogen bond from the amine group (A H 1.842 Å), and as a donor of H-bond to the nitrogen atoms from thiadiazole ring (D‒H A: 1.757 Å and 2.558 Å) (first and second neighboring molecule, respectively). The resorcynyl rings of thiadiazole molecules from neighboring layers (distance of 3.36 Å) interact through π···π stacking interactions. The bond lengths and valence angles in the thiadiazole molecule are similar to those previously measured for structurally similar derivatives (within error bars). The detailed crystallographic data for **1** is given in (Figure S7, Table 3 and Tables S1–S3).

### 2.5. IR (ATR) Spectroscopy

The IR spectra of all of the ligands and their respective Cu(II) and Zn(II) complexes are shown in supplementary material (Figures S8–S10). The high frequency region in the FT-IR spectrum of **1** is dominated by three sharp bands at 3385, 3320, and 3206 cm^−1^. Intramolecular hydrogen bonding between the thiadiazole nitrogen and the phenolic group residing at carbon C7 results in low intensity and significant broadening of the -OH stretching band [21,27]. It is therefore very likely that the band at 3385 cm^−1^ originates from the -OH group present at the C9 carbon, whereas the two remaining sharp bands in this region, namely at 3320 and 3206 cm^−1^, represent the respective symmetrical and asymmetrical stretches of the -NH_2_ group [14,21,27,28]. The thiadiazole ring formation manifests in the presence of a strong and sharp band at approximately 1630 cm^−1^ characteristic of the heterocyclic -C=N- stretches [21]. Another sharp band at 1600 cm^−1^ represents the N-H bending vibrations of the amine [28]. The central part of fingerprint region (1320–1120 cm^−1^) is occupied by a series of sharp bands attributed to the in-plane O-H bending and the C-N stretching vibrations, while the characteristic sharp C-O stretching maxima are present at lower frequency (1270–1120 cm^−1^) [21,27]. A series of weak intensity bands below 660 cm^−1^ originates from the -C-S-C- stretching of the thiadiazole ring as well as out-of-plane vibrations of the hydroxyl O-H bonds [21].

Compared to that of **1**, in the IR spectrum of N-acetylated derivative **2** two relatively sharp bands are present at 3309 and 3158 cm^−1^, most likely as result of the respective phenolic O-H and secondary amide N-H stretching. The carbonyl C=O stretching vibration of the amide moiety is represented by a sharp band at 1680 cm^−1^ [27]. The most significant change in the central part of the fingerprint region is due to the appearance of sharp band at 974 cm^−1^, which may be assigned to the N-H out-of-plane bending vibration. The second most significant change in this region is a band at 623 cm^−1^ suggesting the structural alterations made near the -C-S-C- system of the thiadiazole ring [21].

In the case of fully acetylated compound **3**, only one broad band is present in the region above 3000 cm^−1^, representing most likely the amide N-H stretching. The esterification of both phenolic OH groups manifests in the appearance of high intensity and relatively broad band at 1771 cm^−1^, originating from the C=O stretches of the acetate moieties. Another sharp band at 1695 cm^−1^ represents the C=O stretching of the amide [27]. These features are accompanied with a series of moderate changes in the fingerprint region of the spectrum, with prominent bands present at 1186, 1014, 882, and 672 cm^−1^.

The spectra of complexes **4**–**7** revealed a broad and moderate intensity bands spanning from approximately 3500 to 3000 cm^−1^ suggesting the presence of hydrates, consistent with microanalysis result and AAS (see Section 2.7). This broad band overlapped the remaining characteristic bands which are normally expected to appear in this region, and particularly the N-H stretching. Thus, the involvement of the -NH_2_ group in the complex formation cannot be confirmed by IR spectroscopy. Nevertheless, in all cases a band with maximum intensity between 3450–3300 was still visible suggesting that the peripheral phenolic -OH group of the resorcinol is not involved in binding to the metal center. Compared to the spectrum of free ligand **1**, the corresponding complexes **4** and **5** (Cu(II) and Zn(II) complexes, respectively) revealed a moderate shift of the thiadiazole C=N band, from 1628 cm^−1^ in **1** to 1607 and 1610 cm^−1^ in **4** and **5**, respectively. This points at the ligand–metal interaction occurring via thiadiazole nitrogen, and is further evidenced by numerous changes in the fingerprint region of the spectra, suggesting the metal chelating by both thiadiazole nitrogen and the nearby resorcynyl -OH group (deprotonated). The features relevant to coordination with the metal center in complexes incorporating the N-acetylated ligand **2** (complexes **6** and **7**) are similar to those of compounds **4** and **5**. In addition, the band assigned to the C=O stretching of the amide remained unchanged in **2**, **6**, and **7**, evidencing that this moiety is not involved in coordination. This in turn confirmed the hypothesis that the metal coordination occurs via the phenolic -OH and one of the thiadiazole N atoms. Additional details were noticed in the spectra of Zn(II) complexes **5** and **7**, in which the presence of acetate ion was postulated, namely the broadening of bands present in the region of approximately 1680 cm^−1^. In these complexes, the band originating from the coordinated acetate ion is most likely overlapped with those representing the C=N stretches. The presence of the acetate ion was confirmed by microanalysis (vide infra) for the Zn(II) complexes.

### 2.6. Mass Spectrometry

**Figure 4:**
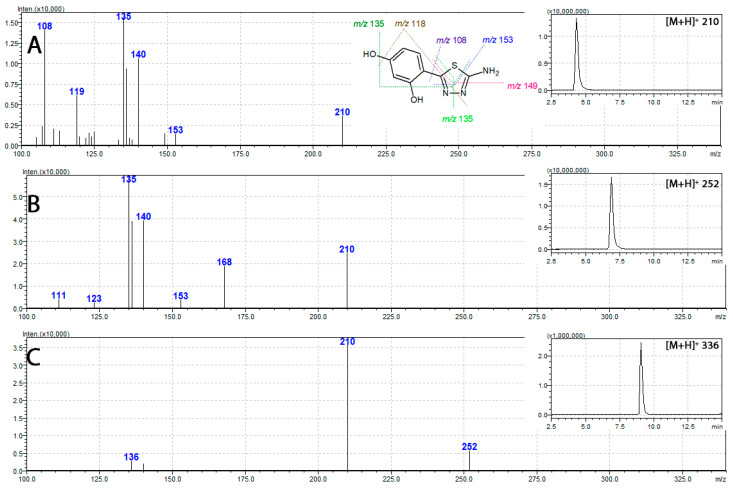
Mass spectra of thiadiazole derivatives **1**, **2**, and **3** (**A**, **B**, and **C**, respectively) together with their respective mass chromatograms (insets).

Regardless of the possibility of free rotation around the resorcynyl-thiadiazole-linking bond the HPLC analysis of each free ligand **1**–**3** revealed only one peak (Figure 4 insets). This suggests that at the aqueous methanolic environment (methanol–water gradient applied for the analysis) only one isomer is present (Figure 3A).

Tandem mass spectrometry showed similar fragmentation patterns for all thiadiazole-derived ligands **1**–**3**. Their respective mass spectra revealed an intensive signal of *m/z* 210, assigned to that of [M + H]^+^, which corresponds to the protonated molecular ion of **1**. The presence of these fragments in spectra of both **2** and **3** (Figure 4) points at the loss of acetyl moieties as an initial fragmentation steps. In more detail, the fragmentation of fully acetylated compound **3** (*m/z* 336 [M + H]^+^) starts with the loss of two O-acetyl moieties and leads to the formation of *m/z* 252 assigned to [M + H]^+^ ion of **2**. The subsequent loss of the amide acetyl results in the formation of *m/z* 210, characteristic of [M + H]^+^ of **1**. Further fragmentation steps in **1**, **2**, and **3**, are identical and rely upon the degradation of *m/z* 210 [M + H]^+^ ion. This involves multiple bond-breaking decomposition steps of the thiadiazole ring with the formation of a number of characteristic signals, namely the *m/z* 108, 135, 149, and 153 [29]. In the case of the metal complexes, the insolubility of their complexes did not allow for their characterization by mass spectrometry.

### 2.7. Elemental Analysis (C,H,N) and Atomic Absorption (AAS) Spectroscopy

The results determined for the free thiadiazole ligands **1**–**3** confirmed their acceptable purities, which were additionally supported by sharp melting point ranges recorded for these compounds as well as the NMR spectra which do not show the presence of additional signals from impurities.

The data obtained from Zn(II) complexes **5** and **7**, suggest the structures incorporating one thiadiazole ligand (**1** and **2**, respectively) and one acetate ion being bound to the central metal, although some controversy may be raised in terms of the number of additional aqua ligands present. Nevertheless the correlation of data obtained from CHN and AAS measurements suggests the presence of two additional water molecules present in both Zn(II) complexes. The presence of acetate counter ion in **5** and **7** is supported by the NMR spectra, which show the characteristic acetate singlet at approximately 1.8 ppm (see Section 2.2), while the presence of additional aqua ligands is supported by the IR spectra, which are characteristic of broad and relatively low intensity bands spanning between 2800 and 3400 cm^−1^ (Figures S9 and S10). These findings point to similar coordination modes in complexes **5** and **7** to those previously obtained in our group [21,30]. Therefore, it is likely that the Zn(II) ion in **5** and **7** is coordinated with thiadiazole ligand via the deprotonated phenolic -OH of the resorcynyl moiety and the neighboring N atom of the thiadiazole ring. Other lone electron pair-containing substituents of the thiadiazole ligands, namely the second phenolic -OH, -NH_2_ group (**5**), amide moiety (**7**), and the remaining two heteroatoms of the thiadiazole ring (N and S) are not involved in binding with Zn(II) ion.

Both CHN and AAS data of the Cu(II) complex **6** fit best with the structure of dihydrate complex, utilizing 2:1 ratio between thiadiazole ligand and the central metal. This corresponds well with structures of the previously reported Cu(II) complexes, incorporating structurally similar thiadiazole ligands [21,30], and is consistent with the lack of acetate ion in the complex structure. Also, the structure of **6** is proposed based on the fact that the thiadiazole ligand **2** lacks the amine moiety. Compared to that of the amine, the amide group present in **2** is characteristic of much lower affinity to the metal ions and hence its involvement in coordination is unlikely.

The data obtained from Cu(II) complex **4** show discrepancies between the calculated and experimentally determined values and due to that fact are less conclusive. More specifically, the comparison of thiadiazole-derived ligands **1** and **2** structures together with the CHN and AAS results from their respective Cu(II) complexes **4** and **6** enable the hypothesis that the formation of **4** may involve the coordination via the -NH_2_ group or the complex formed may be polymeric in nature, which is consistent with the low solubility of the complexes formed. This in turn points at the possibility of much more complicated structure of the complex **4** compared to that of **6**. The more detailed information on structures of the Cu(II) complexes formed and especially the complex **4** would require a more in-depth studies. The microanalysis (C, H, and N) and AAS data together with the proposed formulae of complexes are given in Table 4.

### 2.8. UV–Vis Spectroscopy

The UV–vis spectra of all compounds recorded in methanol are presented in the supplementary material (Figure S8). Although the free thiadiazole ligands **1**–**3** were soluble in methanol, the overall low solubility of complexes **4**–**7** made their analysis more difficult. Nevertheless, the satisfactory quality of the spectra was achieved by preparing the 1 μM solution of each sample.

All compounds have their absorption maxima in the UV region with significant ‘tailing’ of the bands onto the visible range of the spectra. In compounds **1** and **2** the spectra were almost identical, each demonstrating two distinct bands at approximately 290 and 325 nm (Figure S11) and representing the π → π* transitions. Similarly to those of previously reported structures [3,5,31] the high energy and relatively low intensity band originates from the transitions occurring within the phenolic ring, while the lower energy but more intensive band occurs due to the presence of thiadiazole heterocycle, which interacts with the neighboring resorcynyl ring via intramolecular hydrogen bond formed by the -OH group and one of the thiadiazole N atom (See Figure 3B). Moreover, the presence of resorcinol moiety enables the possibility for keto-enol-like tautomerism, which is another structural feature characteristic of this group of compounds and affects the energy of electronic transitions [5]. The acetylating of -NH_2_ group in **1** with the formation of amide moiety in **2** does not alter the chromophore system, hence the lack of significant differences between the spectra of **1** and **2**. In turn, the spectrum of fully acetylated compound **3** is characteristic of only one broad band with the maximum at 293 nm. This obviously is associated with the lack of intramolecular hydrogen bond and steric hindrance to planarity introduced be the acetyl moieties, which affect the charge distribution at the chromophore and result in a shift of the main absorption band towards the higher energy.

The complexation to Cu(II) and Zn(II) ions does not result in any significant shifts of the main absorption band, except in complex **5**, which has its lower energy band shifted from 294 to 342 nm. This points at the structural similarities in all the complexes formed and supports the proposed ligand–metal ratios. Also, the shift of the absorption maximum towards the lower energy region observed in the spectrum of complex **5** is not extraordinary and may be assigned to the charge transfer occurring from the metal to ligand (MLCT), which in turn evidences the complex formation [21]. A significant broadening of the lower energy band is a main feature observed in spectra of all complexes **4**–**7**, suggesting the presence of additional transitions related to charge transfer between the ligand and the metal center [21]. The high energy band at ~290 nm is observed in all spectra of complexes but in all cases its intensity is much lower compared to that of the corresponding free ligand. The bands associated with d → d* transitions in the complexes were not observed, as the concentration at which spectra were recorded was not sufficient to record bands with low molar absorptivity values.

### 2.9. Fluorescence Spectroscopy

The fluorescence emission spectra of all compounds were recorded using 0.25 μM methanolic solutions of compounds obtained by four-fold dilution of 1 μM samples, which were previously used for the UV–vis measurements. The use of 0.25 μM solutions allowed for an elimination of inner filter effect, while offering the possibility for direct comparison of the fluorescence of free ligands as well as metal complexes. All spectra were recorded at identical conditions using the excitation wavelength λ_Ex_ = 290 nm. The results are presented in Figure S12 of supplementary material.

The fluorescence spectra of free ligands **1** and **2** have their respective maxima at 380 and 377 nm, while the emission band in compound **3** shows maximum at 361 nm. These differences depend clearly on the substituents present in compound **1**–**3**, and particularly on the acetyl moieties. In more detail, the compound **1**, which does not have any acetyl group is characteristic of relatively low energy emission (λ_Em(Ex290)_ = 380 nm). Compared to that of compound **1**, the mono-acetylated compound **2** has its emission maximum slightly shifted towards higher energy (λ_Em(Ex290)_ = 377 nm), while the highest energy emission is attributed to the fully acetylated compound 3 (λ_Em(Ex290)_ = 361 nm). These differences may be explained based on the crystallographic data, which evidenced the planar structure of the free ligand **1** (see Section 2.4). In more detail, the planarity of **1** is stabilized by an internal hydrogen bond between the *ortho*-hydroxyl group and the neighboring thiadiazole nitrogen atom. Also, this structural feature is present in **2** suggesting its planarity similar as that of **1**. In terms of the fluorescence emission, the planarity and relative rigidity in **1** and **2** effects in nearly identical wavelengths of their fluorescence maxima, whereas in **3** wherein the planarity is distorted the fluorescence band is positioned at the higher energy wavelength [1,2,32].

In terms of the fluorescence intensities it is worth noting that the fluorescence intensity in **2** is lower compared to that of **3**, but higher than that of **1**. Apparently, the -NH_2_ group in **1** contributes (to some extent) to the non-radiative dissipation of excitation energy, most likely by the formation of hydrogen bonds with environment (solvent or other ligand molecules). Apparently, the acetylating of the amine group in **2** weakens the interactions mentioned, which in turn effects in an increase in fluorescence intensity of the amide derivative formed. This effect is even more pronounced in compound **3** which demonstrated the most intensive fluorescence in the series.

Complexation of **1** and **2** to the metal salts did not effect in any significant shift of the emission maxima of the complexes compared to those of the corresponding free ligands, except the Zn(II) complex **5**, which had its emission maximum shifted to 413 nm. All the remaining complexes showed their emission maxima at approximately 380 nm. It is highly likely that the planarity of ligands **1** and **2** is distorted as result of the replacement of the internal hydrogen bond with the coordination bonds with the central metal. Therefore, in the complex **5**, the shift of emission maximum towards lower energy may be associated with the possibility that upon complexation, the ligand **1** adopts the non-planar geometry. Although this feature clearly evidences the that the complex is formed it cannot be considered as unambiguous evidence for the amine group partaking in coordination to the metal center.

Compared to the spectra of free ligands **1** and **2**, a significant drop in fluorescence intensities of their corresponding complexes was observed. Although the complexation increases the rigidity of the ligand the coordination to metal ion may be attributed to the specific conformation which does not necessarily favors the radiative dissipation of the excitation energy, thus the fluorescence drop observed in the emission spectra of the complexes. In case of the Cu(II) complexes **4** and **6**, the fluorescence intensity drop is most likely associated with the internal conversion from the excited MLCT state [21,33]. Interestingly, also the Zn(II) complexes **5** and **7** demonstrated notably lower fluorescence intensities compared to those of their corresponding free ligands, suggesting the internal conversion as the main relaxation pathway in the series of complexes obtained.

### 2.10. Antioxidant Activity

The antioxidant activity of thiadiazole ligands **1**–**3** was assessed with use of spectrophotometrical method based on a reduction of DPPH^·^ radicals at room temperature. The results obtained were compared with those of TROLOX (Table 5). Due to the solubility issues, the antioxidant activity of the complexes remained undetermined.

All three thiadiazole ligands **1**–**3** demonstrated a significantly lower antioxidant activity compared to that of TROLOX (which equals 1 once expressed as TROLOX equivalent (TE)). Interestingly, the N-acetylated compound **2** showed the highest activity among the compounds tested. Compared to that of 1 the antioxidant potency of **2** was nearly 6-fold greater. The fully acetylated compound **3** showed the highest IC_50_ and compared to **1** and **2** was considered inactive. These findings are consistent with our previous studies [34,35] and with numerous other reports evidencing the polyphenolic moieties as mandatory for high antioxidant potency [36,37,38].

### 2.11. Antibacterial Activity

The antibacterial activities against Gram-negative *Escherichia coli* (ATCC 10536), *Pseudomonas aeruginosa* (ATCC 6538), and Gram-positive *Staphylococcus aureus* (ATCC 15442) bacteria were determined were determined based on a previously reported protocol and expressed as the minimal inhibitory concentration (MIC) [39,40,41]. The commercially available antibiotics ampicillin, tetracycline, kanamycin, and erythromycin were used as the reference standards (Table 6).

The MIC values determined for free ligands **1**–**3** and their corresponding Zn(II) complexes **5** and **7** were in all cases much higher compared to those of the control drugs, pointing at their low antibacterial potency. The highest activities were observed against *S. aureus*, where compounds **1**, **5**, and **7** exhibited the MIC of 0.5 mg/mL. Compared to *S. aureus*, the *E. coli* strain was twice much resistant to the compounds tested, while *p. aeruginosa* remained unaffected.

Regardless the low antibacterial activity of compounds tested a rough estimation regarding their structure–activity relationship can be made. In particular, the acetylating of -NH_2_ and both phenolic -OH groups notably decreases the antibacterial activity, hence the activity of **1** is much higher compared to that of **2** and **3**. Moreover, it is worth-noticing that the complexation of **1** to Zn(II) ions does not seem affecting the activity of the corresponding complex **5**, while the complexation of inactive compound **2** effects in the activity of corresponding complex **7** being comparable to those of the free ligand **1** and the complex **5**. These aspects however would require much more in-depth study in the future.

Aiming at a more accurate assessment of the antibacterial activity of thiadiazole derivatives, a checkerboard method [42,43] was applied for the examination of interactions between model thiadiazole ligand **1**, its corresponding Zn(II) complex **5**, and the commercial drug kanamycin. More specifically, both model compounds **1** and **5** demonstrated a strong synergistic interaction with kanamycin resulting in a notably enhanced activity against *S. aureus* (Table 7). The MIC value of 0.5 µg/mL determined for kanamycin combined with relatively inactive compound **1**, was 8-fold lower compared to that of separately tested kanamycin (3.9 µg/mL) and few orders of magnitude lower compared to that of thiadiazole **1** alone. Interestingly, an identical result was obtained from the mixture of kanamycin with complex **5**, suggesting that the interactions between kanamycin may occur via moieties which are not involved in the formation of metal complex. It is therefore likely that the amine, resorcynyl *para*-phenolic group, and the remaining heteroatoms of the thiadiazole ring may constitute the vehicle for synergistic action with kanamycin against *S. aureus*. Previously, the synergistic antifungal interactions were documented to occur between 1,3,4-thiadiazoles and antifungal agent amphotericin B [12]. In this context, the results obtained in our current work confirm that the synergism with various antibiotics is yet another aspect of the biological activity of 1,3,4-thiadiazoles worth investigating.

## 3. Materials and Methods

All chemicals used for the syntheses were of reagent grade or higher. 2,4-dihydroxybenzaldehyde, thiosemicarbazide, acetic anhydride, phosphorous oxychloride, Trolox (6-Hydroxy-2,5,7,8-tetramethylchromane-2-carboxylic acid), DPPH (2,2-diphenyl-1-picrylhydrazyl), DMSO-d_6_, and MS grade methanol were purchased from Aldrich (Darmstadt, Germany). Concentrated HCl and solid NaOH were purchased from ChemPur (Piekary Śląskie, Poland). Ethanol, methanol, acetonitrile, formic acid, Zn(CH_3_COOH)_2_, and Cu(CH_3_COOH)_2_ were purchased from Avantor (Gliwice, Poland). All solvents were of 99% purity or higher (HPLC grade).

The NMR spectra, were acquired on a Bruker Avance III spectrometer (500 MHz) (Bruker, Coventry, UK), using d_6_-DMSO as solvent. Signal assignments were made using standard techniques including HSQC and HMBC experiments. The infrared spectra were recorded in the region of 4000 cm^−1^ to 450 cm^−1^ on a Thermo Scientific Nicolet iS5 Fourier-transform infrared spectrophotometer equipped with the iD7 ATR adapter (Shimadzu, Kyoto, Japan). The electronic absorption and steady-state fluorescence measurements, antioxidant, and antibacterial assays were performed in 96-well plates on a Tecan Infinite 200 microplate reader (Tecan Austria GmbH, Grödig/Salzburg, Austria). HPLC-ESI-MS analyses were performed on a Shimadzu 8030 ESI-Triple Quad mass spectrometer (Shimadzu, Kyoto, Japan). All HPLC-MS analyses were performed in positive ion mode. The HPLC solvents gradient was 40% B in A at 0 min to 90% B in A at 15 min (A: 2% *v/v* formic acid in water; B: methanol). Helium (He) was used as a collision gas during collision-induced (CID) MS/MS experiments and collision energy (CE) was set at −35 V. The X-ray data collection for single crystal (T = 120K) was carried out on a SuperNova diffractometer (Oxford Diffraction, Oxford, UK), with micro-focusing source of CuKα radiation. Indexing, integration, and scaling was done using CrysAlis RED software [44]. The structure was solved with direct methods and then successive least-square refinements were carried out, based on the full-matrix least-squares on F^2^ using the SHELX program package [45]. All heavy atoms were refined anisotropically. Hydrogen atoms were fitted isotropically with geometry idealized positions except those forming intermolecular H-bonds. Table S1 includes experimental details for a measured single crystal. Presented structure has been deposited in the CCDC with no. 1845297. Melting point values were recorded on a Stuart SMP20 apparatus within the range of 25–300 °C, and were uncorrected.
*Synthesis of 2-amino-5-(2,4-dihydroksyphenyl)-1,3,4-thiadiazole*(**1**)

2,4-dihydroxybenzoic acid (5.00 g, 32.00 mmol) was suspended in POCl_3_ (15 mL) and stirred at room temperature for 20 min. Thiosemicarbazide (2.95 g, 32.00 mmol) was then added and the reaction mixture was refluxed at 75 °C and stirred for 12 hours. The thick, yellow slurry that formed was cooled down to 30 °C followed by quenching the excess POCl_3_ by slow addition of small aliquots of water. The mixture was then refluxed at 105 °C for 5 h and then it was cooled down to ambient temperature and the pH was then brought to 8.5 with saturated NaOH. The precipitate formed was filtered off, washed with water, and allowed to dry in air. The dry solid was washed thoroughly with methanol and the solution was evaporated to dry under reduced pressure yielding compound **1**. Single crystals suitable for X-ray diffraction were grown in ethanol. Yield: 5.32 g (79%); C_8_H_7_N_3_O_2_S (209.22 g/mol); calc: C 45.93, H 3.37, N 20.08%, found: C 42.96, H 3.30, N 17.84%; M.P.: 252–255 °C; ^1^H-NMR (DMSO): δ = 10.91 ppm (s, 1H, H9, (-OH)), 9.84 (s, 1H, H7, (-OH)), 7.53 (d, 1H, H11, *J* = 8.51 Hz), 7.15 (s, 2H, H12 (-NH_2_)), 6.38 (d, 1H, H8, *J* = 2.28 Hz), 6.36 (dd, 1H, H10, *J_1_* = 8.51, *J_2_* = 2.28 Hz); ^13^C-NMR (DMSO): 167.84 ppm (C5), 160.22 (C9), 156.43 (C7), 156.03 (C2), 129.20 (C11), 108.99 (C6), 108.35 (C10), 102.99 (C8); IR (ATR): 3385, 3320, 3206, 2656, 2584, 1628, 1604, 1530, 1514, 1472, 1317, 1268, 1174, 1125,1057, 983, 967, 831, 761, 655, 459 cm^−1^; UV-Vis (MeOH): λ_1_ = 294, λ_2_ = 324 nm; Fluorescence (MeOH): λ_Em(Ex290)_ = 380 nm.
*Synthesis of 2-acetamido-5-(2,4-dihydroksyphenyl)-1,3,4-thiadiazole*(**2**)

Compound **1** (1.00 g, 4.78 mmol) was refluxed in the mixture of acetic anhydride (10 mL) and water (4 mL) for 6 hours. The reaction mixture was then cooled to ambient temperature and the solid was filtered off, washed with water, and dried. The product was recrystallized from ethanol yielding 0.86 g (72%) of 2. Yield: 0.91 g (72%); C_10_H_9_N_3_O_3_S (251.26 g/mol); calc: C 47.80, H 3.61, N 16.72%, found: C 46.34, H 3.41, N 16.21%, M.P.: >300 °C; ^1^H-NMR (DMSO): δ = 12,32 (s, 1H, H12 (-NH-)), 10.90 (s, 1H, H7(-OH)), 9.92 (s, 1H, H9 (-OH)), 7.91 (d, 1H, H11, *J* = 8.74 Hz), 6.45 (d, 1H, H8, *J* = 2.30 Hz), 6.40 (dd, 1H, H10, *J_1_* = 8.74, *J_2_* = 2.30 Hz), 2.18 (s, 3H, H14); ^13^C-NMR (DMSO): 168.79 ppm (C2), 160.94 (C9), 158. 89 (C13), 158. 87 (C5), 156.35 (C7), 129.03 (C11), 109.15 (C6), 108.62 (C10), 102.88 (C8), 22.88 (C14); IR (ATR): 3309, 3158, 2885, 2791,1680,1626, 1597,1557, 1527, 1483, 1415, 1310, 1217, 1180, 1129, 974, 841, 804, 709, 681, 659, 623, 518, 467 cm^−1^; UV-Vis (MeOH): λ_1_ = 292, λ_2_ = 324 nm; Fluorescence (MeOH): λ_Em(Ex290)_ = 377 nm.
*Synthesis of 2-acetamido-5-((phenyl-2,4-diacetate)-yl)-1,3,4-thiadiazole*(**3**)

Compound **1** (0.36 g, 1.70 mmol) was suspended in acetic anhydride (10 mL) and three drops of concentrated H_2_SO_4_ was added. The mixture was refluxed for 6 h and then cooled to ambient temperature and the solid was filtered off, washed with water, and allowed to dry in air. The crude product was recrystallized from ethanol yielding 0.48 g (58%) of **3**. Yield: 0.48 g (58%); C_14_H_13_N_3_O_5_S (335.33 g/mol); calc: C 50.15, H 3.91, N 12.53%, found: C 49.02, H 3.66, N 12.50%; M.P.: 269–271 °C; ^1^H-NMR (DMSO): δ = 12,68 (s, 1H, H12 (-NH-)), 8.23 (d, 1H, H11, *J* = 8.56 Hz), 7.29 (d, 1H, H8, *J* = 2.27 Hz), 7.26 (dd, 1H, H10, *J_1_* = 8.56, *J_2_* = 2.27 Hz), 2.38 (s, 3H, H18), 2.31 (s, 3H, H16), 2.22 (s, 3H, H14); ^13^C-NMR (DMSO): 169.33 ppm (C17), 169.24 (C2), 168. 97 (C15), 160.03 (C13), 155.92 (C5), 152.53 (C9), 147.89 (C7), 129.72 (C11), 121.36 (C6), 121.02 (C10), 118.21 (C8), 22.86 (C14), 21.68 (C18), 21.31 (C16); IR (ATR): 3154, 2899, 2782, 1771, 1695, 1612, 1588, 1563, 1504, 1440, 1336, 1321, 1213, 1185, 1150, 1117, 1105, 1014, 991, 900, 882, 820, 686, 672, 609, 551, 473 cm^−1^; UV-Vis (MeOH): λ_1_ = 293 nm; Fluorescence (MeOH): λ_Em(Ex290)_ = 361 nm.
*Synthesis of Zn(II) and Cu(II) complexes*(**4**–**7**)

The Zn(II) complexes were synthesized according to the previously reported procedure [21]: Typically, the free ligand (1.70 mmol) was dissolved in a hot mixture of 30 mL MeOH and H_2_O (1:1 v/v) and equimolar amount of Zn(II) acetate monohydrate was added. The mixture was heated under reflux for 6 h and cooled down to the ambient temperature. A fine solid formed was then collected with the centrifuge, rinsed with water, and dried. The crude product was recrystallized from methanol. The syntheses of Cu(II) complexes were carried out in a similar manner, except that Cu(II) acetate monohydrate (0.85 mmol) was used. The compound **1** was used as a substrate in the synthesis of complexes **4** and **5**, while the compound **2** was applied for the synthesis of **6** and **7**.

(**4**) Yield: 38%; C_16_H_16_CuN_6_O_6_S_2_ (516.01 g/mol); calc: C 37.24, H 3.13, N 16.29, Cu 12.31%, found: C 26.85, H 2.34, N 10.69, Cu 24.67%; M.P.: >300 °C; IR (ATR): 3447, 3311, 1607, 1553, 1475,1428,1239,1187, 1172, 1128, 1080, 994, 979, 825, 728, 684, 619, 456 cm^−1^; UV-Vis (MeOH): λ_1_ = 322 nm; Fluorescence (MeOH): λ_Em(Ex290)_ = 380 nm.

(**5**) Yield: 46%; C_10_H_13_N_3_O_6_SZn (368.67 g/mol); calc: C 32.58, H 3.55, N 11.40, Zn 17.73%, found: C 30.98, H 2.74, N 12.02, Zn 16.93%; M.P.: >300 °C; IR (ATR): 3413, 3233, 1610, 1558, 1477, 1221, 1178, 1128, 1085, 992, 977, 886, 835, 675, 604, 451cm^−1^; UV-Vis (MeOH): λ_1_ = 294, λ_2_ = 342 nm; Fluorescence (MeOH): λ_Em(Ex290)_ = 413 nm.

(**6**) Yield: 41%; C_20_H_20_CuN_6_O_8_S_2_ (600.08 g/mol); calc: C 40.03, H 3.36, N 14.01, Cu 10.59%, found: C 37.67, H 2.95, N 12.90, Cu 12.04%; M.P.: >300 °C; IR (ATR): 3159, 2910, 2770, 1681, 1598, 1540, 1476, 1413, 1368, 1311, 1219, 1179, 1142, 1130, 976, 833, 798, 760, 706, 682, 625, 603, 551, 517 cm^−1^; UV-Vis (MeOH): λ_1_ = 292, λ_2_ = 322 nm; Fluorescence (MeOH): λ_Em(Ex290)_ = 374 nm.

(**7**) Yield: 42%; C_12_H_15_N_3_O_7_SZn (410.71 g/mol); calc: C 35.09, H 3.68, N 10.23, Zn 15.92%, found: C 37.01, H 3.02, N 11.55, Zn 13.21%; M.P.: >300 °C; IR (ATR): 3033, 2893, 2770, 1681, 1625, 1598, 1559, 1480, 1418, 1371, 1329, 1312, 1220, 1180, 1130, 1043, 991, 975, 842, 804, 758, 681, 631, 519, 468 cm^−1^; UV-Vis (MeOH): λ_1_ = 293, λ_2_ = 328 nm; Fluorescence (MeOH): λ_Em(Ex290)_ = 372 nm.

### 3.1. Antioxidant Assay

To a transparent, 96-well plate an increasing concentrations of individual ligands solutions were added. Next, 40 μL of 1.0 mM DPPH^·^ radicals methanolic solution was applied. The concentration of each ligand (**1**–**3**) was set so that the decrease of absorption intensity of DPPH˙ radicals solution at λ_max_ 519 nm after 30 minutes of the reaction kept in the dark was in the range of 10–90% of its initial value. The total volume of all samples was 200 μL. The plate was shaken for 10 s on the reader shaker to obtain homogeneous solutions and then the absorbance measurement at λ_max_ 519 nm started. All data were collected for 30 min at 25 °C. The final values were the average of five exposures of the sample to a beam of light. Each sample was repeated three times in independent experiments.

### 3.2. Antibacterial Activity Assay

The bacterial strains were incubated in Mueller–Hinton Broth medium at 37 °C over 24 h in aerobic conditions. The number of cells in the suspension was adjusted to that of 0.5 McFarland standard, which was an equivalent of 108 colony-forming units (CFU). The antibacterial activities of thiadiazole ligands and their Zn(II) complexes were determined as minimal inhibitory concentration (MIC) using broth dilution method. All experiments were performed based on the standard protocol [39,40]. The experiments were carried out in 96-well plate and the thiadiazole derivatives were tested within the concentration range of 7.9 µg/mL to 1 mg/mL. All experiments were run in triplicate.

Additionally, the possibility of synergistic antibacterial action was assessed with use of the checkerboard method, in which the compounds **1**–**3**, **5**, and **7** were combined with kanamycin and the mixtures were examined for their activity against *S. aureus.*

## 4. Conclusions

In conclusion, a series of 2-amino-5(2,4-hydroxyphenyl)-1,3,4-thiadiazole-derived homologues were synthesized with use of classical methods and their ability to form metal complexes with Zn(II) and Cu(II) ions was examined. All compounds were isolated with satisfactory purities and good yields. The structures of all compounds were confirmed using a number of spectroscopic techniques including the UV–vis, fluorescence, IR, NMR (^1^H, ^13^C, HSQC, HMBC), HPLC-MS, AAS, and CHN analysis. Moreover, the structure of model thiadiazole ligand 1 was examined in detail with use of single crystal X-ray diffraction technique. The structural features of thiadiazole derivatives isolated were discussed for their metal binding site and especially for the involvement of the amino group in the metal complex formation. The results obtained suggested that the metal-binding pocket in thiadiazole ligands is formed via the neighboring *ortho*-phenolic group and one of the thiadiazole nitrogen atoms. The structures of complexes Zn(II) and Cu(II) formed differ in their metal:thiadiazole ratios, which in Zn(II) complexes is 1:1, while in Cu(II) it is 1:2. Moreover, in Zn(II) complexes, an acetate ion is present as an additional ligand. Furthermore, all complexes are most likely form hydrates utilizing two water molecules as additional ligands partaking in the coordination to the central metal. The results obtained suggest that the metal complex formation does not occur via the -NH_2_ group present in the ligand structure. This enables the possibility for relative ease in structural modifications of the thiadiazole ligands isolated, while keeping the metal binding site unaltered. This structural feature appears particularly useful once considered in terms of sparing solubility of the complexes.

Low solubility in aqueous media has a negative influence on the antibacterial activity and manifests in the fact that all thiadiazole derivatives are characteristic of MIC values notably higher compared to those of commercially used antibiotics. On the other hand, one of derivatives in the series exhibited a strong synergistic effect with kanamycin against *S. aureus* species, making the prospect of further investigation on that field particularly attractive. In this context, an alteration of the amine moiety towards the products characteristic of an increased aqueous solubility would certainly enhance the biological activity of both free ligands and their metal complexes. Such modifications are undoubtedly necessary, especially in terms of potential applicability of thiadiazole ligands as novel anti-neurodegenerative agents. Therefore, the future work would involve a modification of the amine moiety of the thiadiazole ligands towards increased aqueous solubility, and their potential applicability as novel therapeutic agents.

## Figures and Tables

**Table 1:** ^1^H-NMR signals of the 1,3,4-thiadiazole derivatives 1–3.

Thiadiazole Serivative	^1^H-NMR Signals: δ (ppm) *, Multiplicity **, and J Value (Hz)								
	H7	H8	H9	H10	H11	H12	H14	H16	H18
**1**	10.91, *s* (-OH)	6.38, *d*, *J* = 2.3	9.84, *s*, (-OH)	6.36, *dd*, *J_1_* = 8.5, *J_2_* = 2.3	7.53, *d*, *J* = 8.5	7.15, *s*, (-NH_2_)	-	-	-
**2**	10.90, *s* (-OH)	6.45, *d*, *J* = 2.3	9.92, *s*, (-OH)	6.40, *dd*, *J_1_* = 8.7, *J_2_* = 2.3	7.91, *d*, *J* = 8.7	12.32, *s*, (-NH-)	2.18, *s*	-	-
**3**	-	7.29, *d*, *J* = 2.3	-	7.26, *dd*, *J_1_* = 8.5, *J_2_* = 2.3	8.2, *d*, *J* = 8.5	12.68, *s*, (-NH-)	2.22, *s*	2.31, *s*	2.38, *s*

* = numbering system of atoms shown in Figure 1; ** s = singlet, d = doublet, dd = double doublet.

**Table 2:** ^13^C-NMR data of the 1,3,4-thiadiazole derivatives 1–3.

Thiadiazole Derivative	^13^C-NMR Signals (ppm) *													
	C2	C5	C6	C7	C8	C9	C10	C11	C13	C14	C15	C16	C17	C18
**1**	167.84	156.03	108.98	156.43	103.00	160.22	108.35	129.20	-	-	-	-	-	-
**2**	168.79	158.87	109.15	156.35	102.88	160.94	108.62	129.03	158.89	22.88	-	-	-	-
**3**	169.24	155.92	121.36	147.89	118.21	152.53	121.02	129.72	160.03	22.87	168.96	21.31	169.33	21.68

* = numbering system shown in Figure 1.

**Table 3:** Crystallographic parameters and details of refinement for the measured crystal of compound **1**. The labels are related to the Figure 3A.

Parameter	Value
Molecular formula	C_8_H_7_N_3_O_3_S
Temperature (K)	120(2)
Crystal system	monoclinic
Space group	I 2/a
*a* (Å)	13.421(2)
*b* (Å)	7.1590(10)
*c* (Å)	18.454(2)
*α* (°)	90
*β* (°)	100.79(2)
*γ* (°)	90
*V* (Å^3^)	1741.73
*Z*	8
Calculated density (g cm^−3^)	1.596
Absorption coefficient (mm^−1^)	3.133
*F* (000)	864
Completeness	97%
θ range for data collection (°)	4.88–76.15
Index ranges	−13 ≤ h ≤ 16 −8 ≤ k ≤ 7 −22 ≤ l ≤ 15
Reflections collected/unique	6137/3789 (R_int_ = 0.0353)
Observed/restraints/parameters	1761/0/151
Goodness-of-fit on *F*^2^	1.217
Final R indices (I > 2 sigma(I))	R1 = 0.0551 wR2 = 0.1679
R indices (all data)	R1 = 0.0709 wR2 = 0.2225
Largest diff. peak and hole (e Å^−3^)	0.5/−0.7
CCDC number	1845297

**Table 4:** Microanalysis (C,H,N), AAS, molecular weight, and chemical formulae of thiadiazole derivatives **1**–**7**.

Compound No	%C		%H		%N		%M *		Mw ** (g/mol)	Chemical Formula **
	Calc	Found	Calc	Found	Calc	Found	Calc	Found		
**1**	45.93	42.96	3.37	3.30	20.08	17.84	-	-	209.22	C_8_H_7_N_3_O_2_S
**2**	47.80	46.34	3.61	3.41	16.72	16.21	-	-	251.26	C_10_H_9_N_3_O_3_S
**3**	50.15	49.02	3.91	3.66	12.53	12.50	-	-	335.33	C_14_H_13_N_3_O_5_S
**4**	37.24	26.85	3.13	2.34	16.29	10.69	12.31	24.67	516.01	C_16_H_16_CuN_6_O_6_S_2_
**5**	32.58	30.98	3.55	2.74	11.40	12.02	17.73	16.93	368.67	C_10_H_13_N_3_O_6_SZn
**6**	40.03	37.67	3.36	2.95	14.01	12.90	10.59	12.04	600.08	C_20_H_20_CuN_6_O_8_S_2_
**7**	35.09	37.01	3.68	3.02	10.23	11.55	15.92	13.21	470.71	C_12_H_15_N_3_O_7_SZn

* M = Metal. ** Proposed based on CHN and AAS analysis.

**Table 5:** Antioxidant activity and IC_50_ values of free thiadiazole ligands **1**–**3** and TROLOX after 30 minutes of reaction with DPPH^·^ radicals (± standard deviation (SD) calculated from experiments carried out in triplicate).

Compound	mM Antioxidants/mM TE		IC_50_ (mM)	
**1**	0.08	±0.00	0.60	±0.01
**2**	0.35	±0.03	0.13	±0.00
**3**	0.41 × 10^−3^	±0.02 × 10^−3^	126.78	±2.25
**TROLOX**	1.00	±0.01	0.05	±0.00

**Table 6:** MIC values of thiadiazole derivatives against *S. aureus*, *E. coli*, and *p. aeruginosa* referenced to commercially used antibiotics.

Compound Bacteria	MIC (μg/mL) (±0.01)								
	1	2	3	5	7	Ampicillin	Tetracycline	Erythromycin	Kanamycin
*Staphylococcus aureus*	500	-	-	500	500	1.9	0.1	250	3.9
*Escherichia coli*	1000	-	-	1000	1000	31.2	0.5	62.5	7.8
*Pseudomonas aeruginosa*	-	-	-	-	-	62.5	500	250	-

**Table 7:** Synergistic effects against *S. aureus* demonstrated by 1 and 5 upon their combination with kanamycin.

Compound	MIC ^a^ (µg/mL) (±0.01)	Combination	MIC ^b^ (µg/mL) (±0.01)	FIC ^c^
**1**	500	**1**/kanamycin	125	0.375
kanamycin	3.9		0.5	
**5**	500	**5**/kanamycin	125	0.375
kanamycin	3.9		0.5	

^a^ MIC = value determined in an individual compound (1, 5, and kanamycin); ^b^ MIC = value determined in a combination of two compounds (1 or 5 with kanamycin); ^c^ FIC = fractional inhibitory concentration [40].

## Supplementary Materials

Can be found at https://www.mdpi.com/1422-0067/21/16/5735/s1. Table S1. Atomic distances from crystallographic structure. The labels are related to the Figure S7. Table S2. Interatomic angles from crystallographic structure. The labels are related to the Figure S7. Table S3. Intermolecular contacts. The labels are related to the Figure S7. Figure S1. 1H-NMR Spectrum of thiadiazole derivative **1**. Figure S2. 1H-NMR Spectrum of thiadiazole derivative **2**. Figure S3. 1H-NMR Spectrum of thiadiazole derivative **3**. Figure S4. 13C-NMR Spectrum of thiadiazole derivative **1**. Figure S5. 13C-NMR Spectrum of thiadiazole derivative **2**. Figure S6. 13C-NMR Spectrum of thiadiazole derivative **3**. Figure S7. Crystal Structure of **1** (Atom numeration according to the cif file). Figure S8. Comparison of the IR Spectra of thiadiazole derivatives **1** (top), **2** (middle), and **3** (bottom). Figure S9. Comparison of the IR Spectra of thiadiazole **1** (top), and its corresponding Cu(II) and Zn (II) complexes **4** and **5** (middle and bottom, respectively). Figure S10.Comparison of the IR Spectra of thiadiazole **2** (top), and its corresponding Cu(II) and Zn (II) complexes **6** and **7** (middle and bottom, respectively). Figure S11. UV-Vis spectra of the thiadiazole derivatives **1**–**3** and their corresponding Cu(II) and Zn(II) complexes **4**–**7**. Figure S12. Steady state fluorescence spectra of the thiadiazole derivatives **1**–**3** and their corresponding Cu(II) and Zn(II) complexes **4**–**7** The spectra recorded using the excitation wavelength λ_Ex_ = 290 nm.
