# Supplementary material for: Structural Features of 1,3,4-Thiadiazole-Derived Ligands and Their Zn(II) and Cu(II) Complexes Which Demonstrate Synergistic Antibacterial Effects with Kanamycin
Source: Int J Mol Sci. 2020 Aug 10;21(16):5735. doi: 10.3390/ijms21165735 (PMC7461131; doi:10.3390/ijms21165735)
Supplement: Supplementary file 1 [file ijms-21-05735-s001.pdf]

International Journal of
Molecular Sciences

MDPI

Structural features of 1,3,4-thiadiazole-derived ligands and their Zn(II) and Cu(II) complexes which demonstrate synergistic antibacterial effects with kanamycin

Dariusz Karcz [1,*], Arkadiusz Matwijczuk[2], Daniel Kamiński [3], Bernadette Creaven[4], Ewa Ciszkowicz[5], Karolina Lecka-Szlachta[5] and Karolina Starzak [1]

[1] Department of Analytical Chemistry (C1), Faculty of Chemical Engineering and Technology, Cracow University of Technology, Warszawska 24, 31-155 Kraków, Poland; dariusz.karcz@pk.edu.pl (D.K), karolina.starzak@pk.edu.pl (K.S)
[2] Department of Biophysics, University of Life Sciences in Lublin, Akademicka 13, 20-950 Lublin, Poland; arkadiusz.matwijczuk@up.lublin.pl
[3] Department of General and Coordination Chemistry and Crystallography, Institute of Chemical Sciences, Maria Curie-Sklodowska University in Lublin, pl. Marii Curie-Skłodowskiej 2, 20-031 Lublin, Poland; dkami@umcs.pl
[4] School of Chemical and Pharmaceutical Sciences, Technological University Dublin, Kevin St., Dublin 2, Ireland; bernie.creaven@tudublin.ie
[5] Department of Biotechnology and Bioinformatics, Faculty of Chemistry, Rzeszow University of Technology, 35-959 Rzeszów, Poland; eciszkow@prz.edu.pl (E.C), szlachta@prz.edu.pl (K.L.S.)

* Correspondence:dariusz.karcz@pk.edu.pl; Tel.: +48(12)6282177 (D.K.)

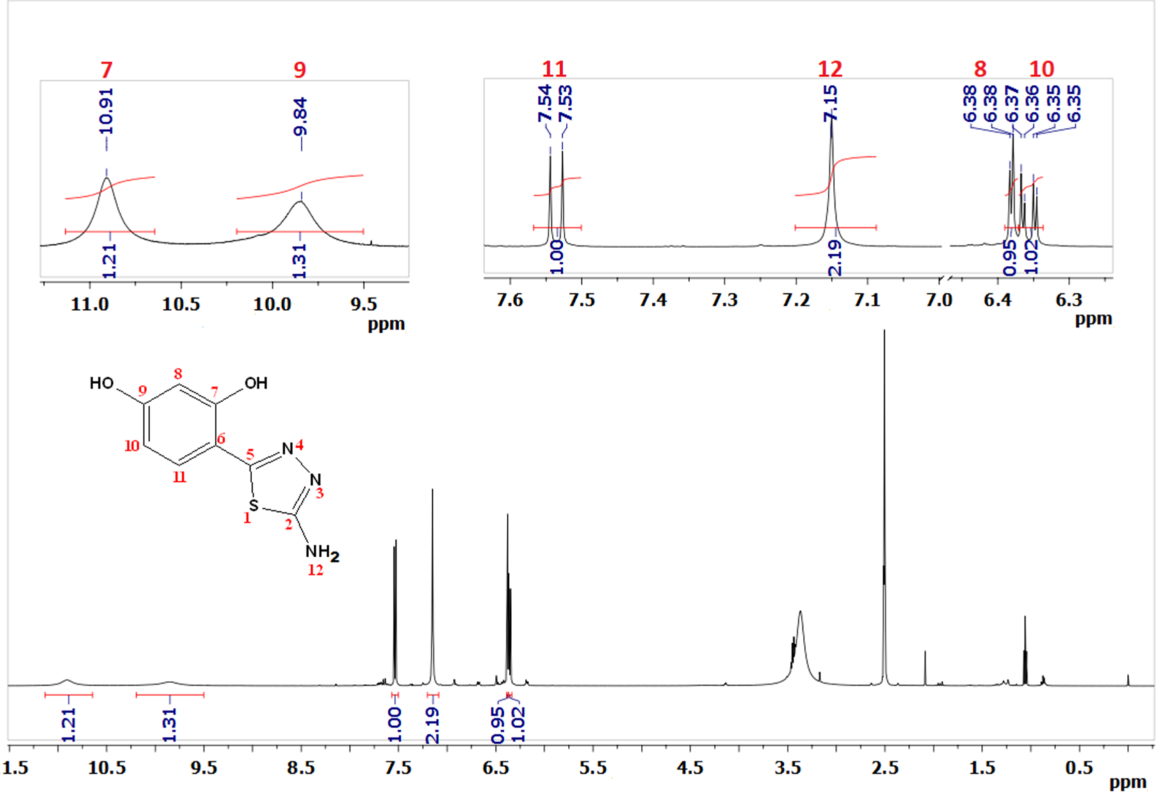

Figure 1. 1H-NMR Spectrum of thiadiazole derivative **1.**

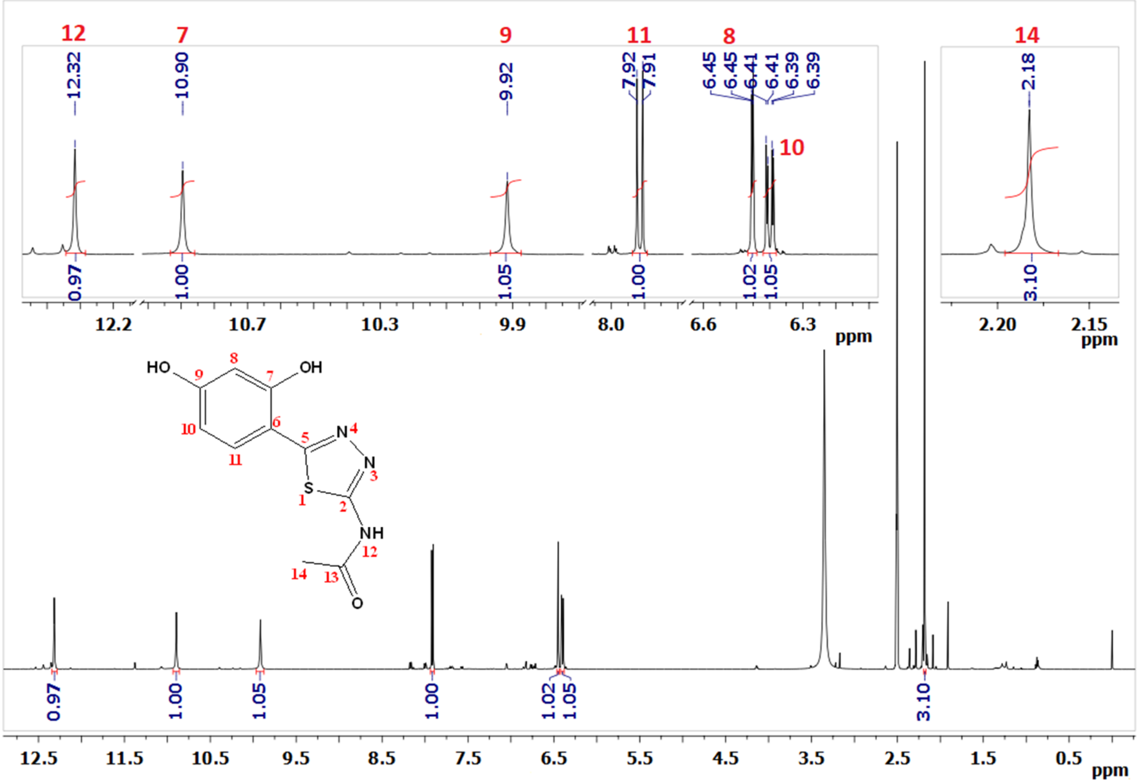

Figure 2. 1H-NMR Spectrum of thiadiazole derivative **2.**

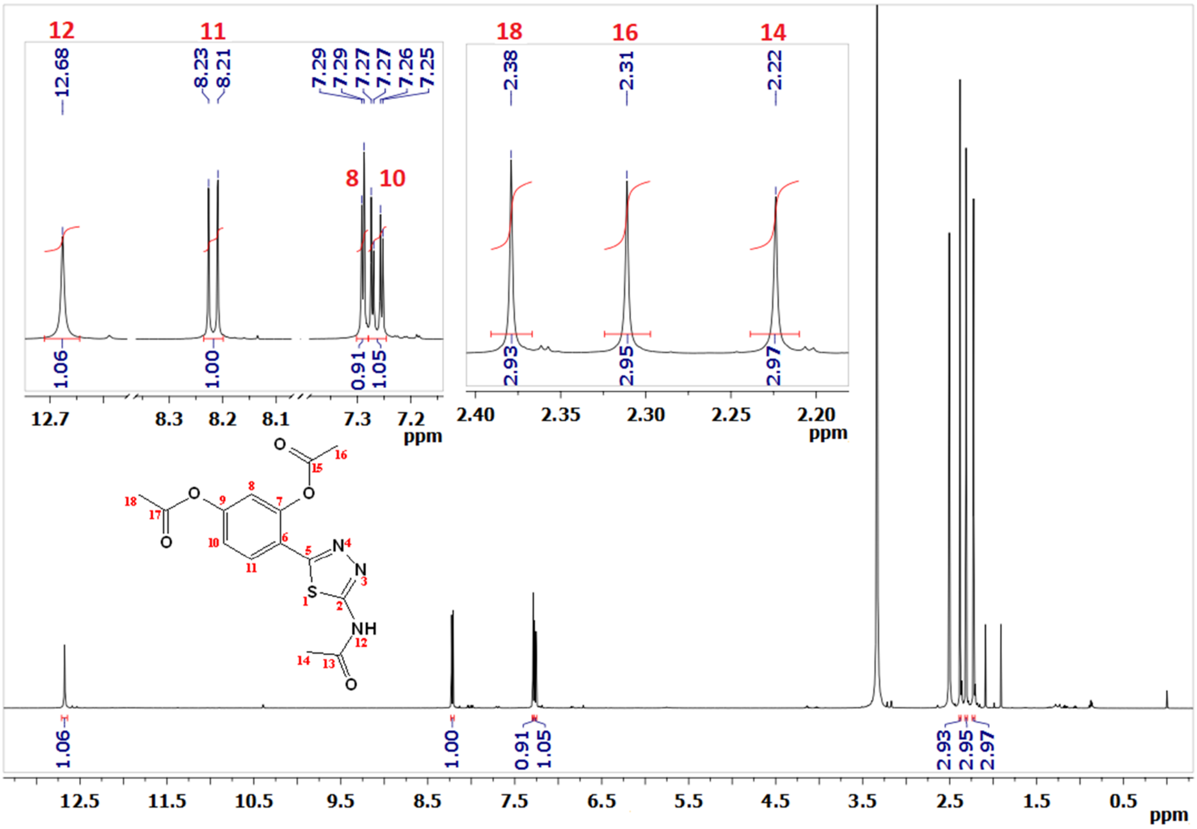

Figure 3. 1H-NMR Spectrum of thiadiazole derivative **3.**

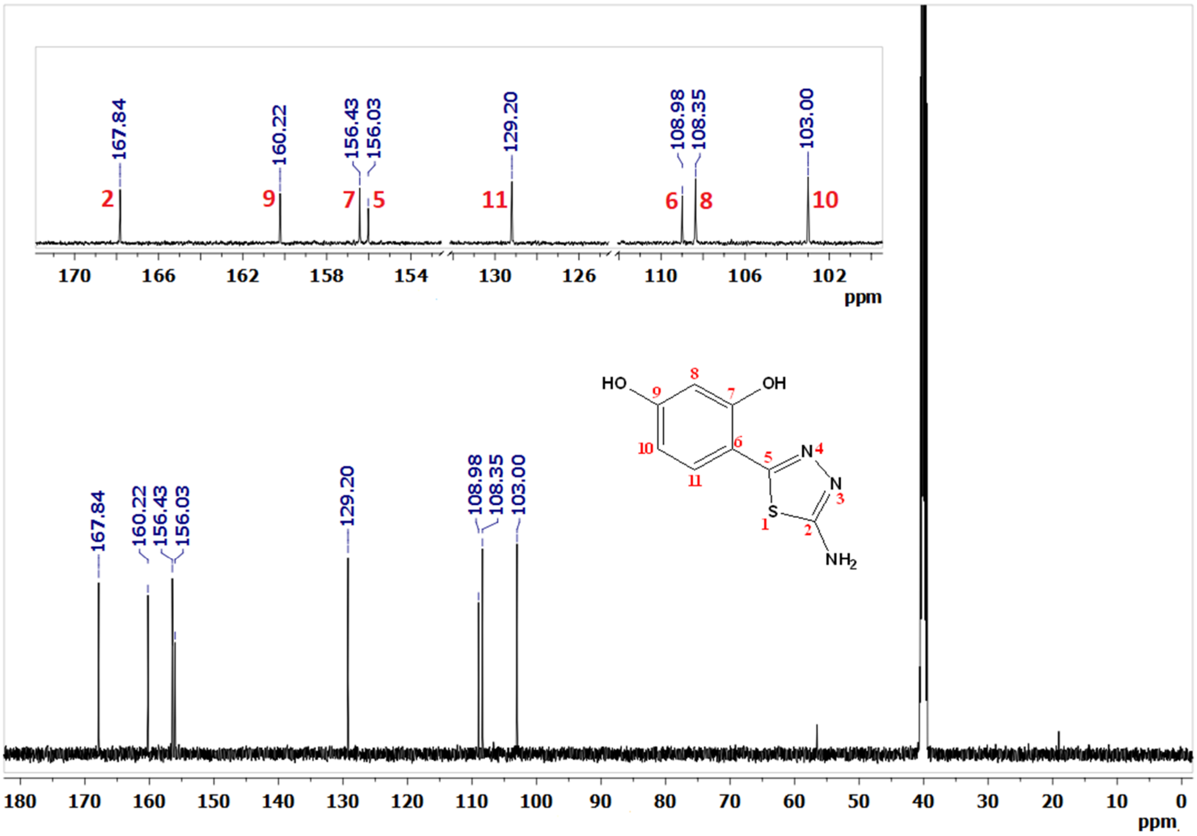

Figure 4. 13C-NMR Spectrum of thiadiazole derivative **1.**

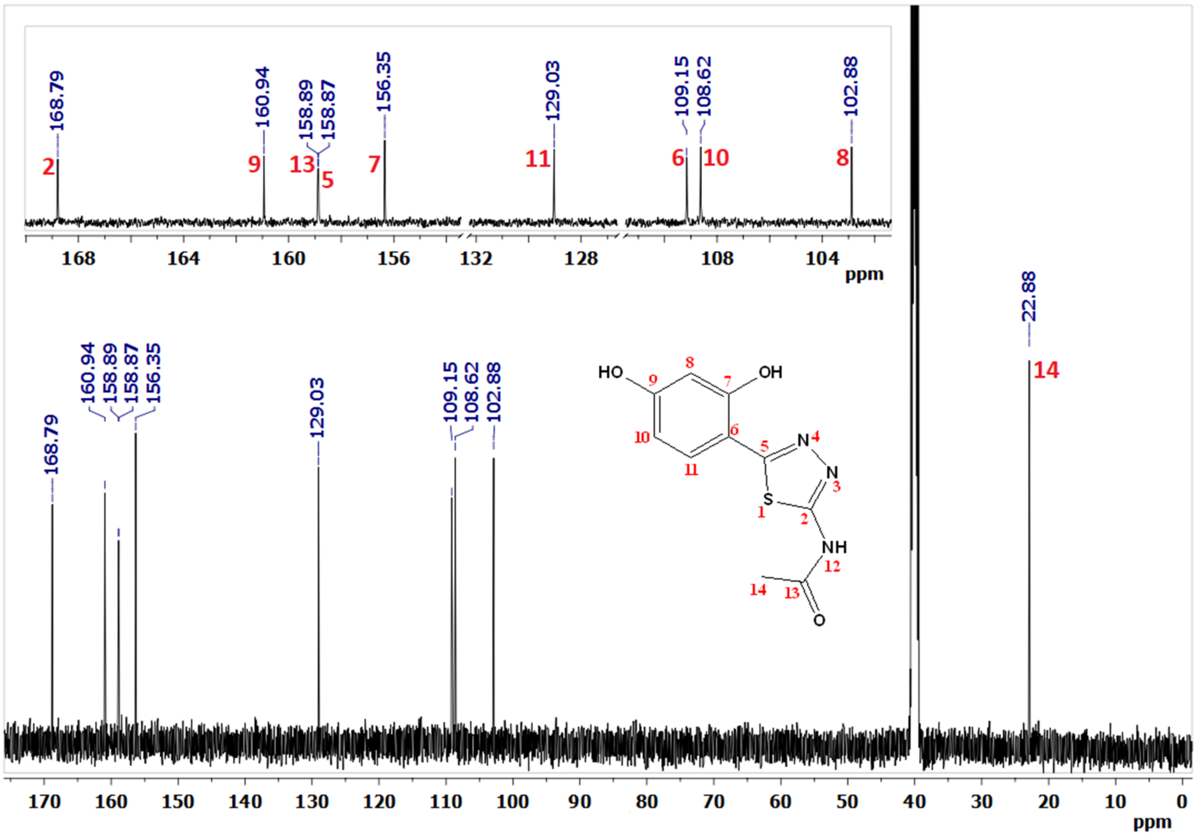

Figure 5. 13C-NMR Spectrum of thiadiazole derivative **2.**

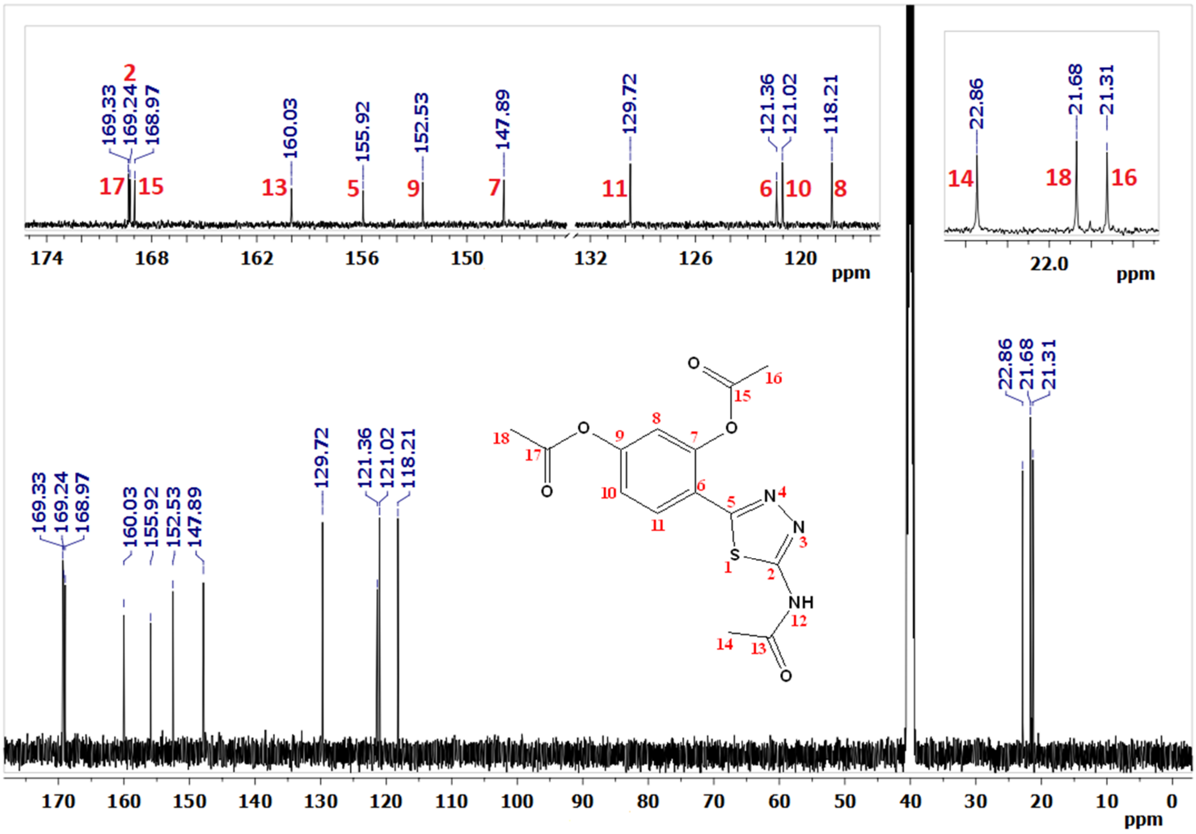

Figure 6. 13C-NMR Spectrum of thiadiazole derivative **3.**

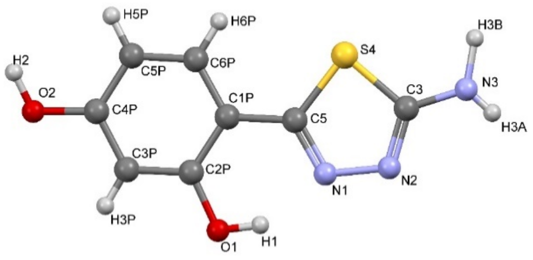

Figure 7. Crystal Structure of **1** (Atom numeration according to the cif file).

Table 1. Atomic distances from crystallographic structure. The labels are related to the Figure S7.

Atom1	Atom2	Length
C3	N2	1.313(5)
C3	N3	1.345(5)
C3	S4	1.740(4)
C1P	C5	1.451(4)
C1P	C2P	1.410(4)
C1P	C6P	1.395(5)
C5	N1	1.315(5)
C5	S4	1.743(4)
C2P	C3P	1.381(4)
C2P	O1	1.362(4)
C3P	C4P	1.396(5)
C3P	H3P	0.90(4)
C4P	C5P	1.400(4)
C4P	O2	1.354(4)
C5P	H5P	0.950
C5P	C6P	1.374(5)
C6P	H6P	0.95(4)
N2	N1	1.380(4)
N3	H3A	0.75(6)
N3	H3B	1.10(6)
O1	H1	0.91(5)
O2	H2	0.93(6)

Table 2. Interatomic angles from crystallographic structure. The labels are related to the Figure S7.

Atom1	Atom2	Atom3	Angle
N2	C3	N3	123.6(3)
N2	C3	S4	114.1(3)
N3	C3	S4	122.3(3)
C5	C1P	C2P	120.5(3)
C5	C1P	C6P	121.9(3)
C2P	C1P	C6P	117.6(3)
C1P	C5	N1	122.7(3)
C1P	C5	S4	124.9(2)
N1	C5	S4	112.4(2)
C1P	C2P	C3P	120.9(3)
C1P	C2P	O1	121.7(3)
C3P	C2P	O1	117.4(3)
C2P	C3P	C4P	120.1(3)
C2P	C3P	H3P	118(3)
C4P	C3P	H3P	122(3)
C3P	C4P	C5P	119.7(3)
C3P	C4P	O2	117.6(3)
C5P	C4P	O2	122.7(3)
C4P	C5P	H5P	120.2
C4P	C5P	C6P	119.4(3)
H5P	C5P	C6P	120.4
C1P	C6P	C5P	122.3(3)
C1P	C6P	H6P	119(2)
C5P	C6P	H6P	118(2)
C3	N2	N1	111.8(3)
C5	N1	N2	114.3(3)
C3	N3	H3A	120(4)
C3	N3	H3B	117(3)
H3A	N3	H3B	122(5)
C2P	O1	H1	108(4)
C4P	O2	H2	116(3)
C3	S4	C5	87.5(2)

Table 3. Intermolecular contacts. The labels are related to the Figure S7.

Atom1	Atom2	Length	Symm. operation
N1	H1	1.778	x,y,z
O2	H3B	1.842	-1/2+x,-1/2+y,-1/2+z
O1	H3A	2.236	1-x,-1/2+y,1.5-z
H2	N2	1.757	x,1/2-y,-1/2+z
H2	N1	2.558	x,1/2-y,-1/2+z

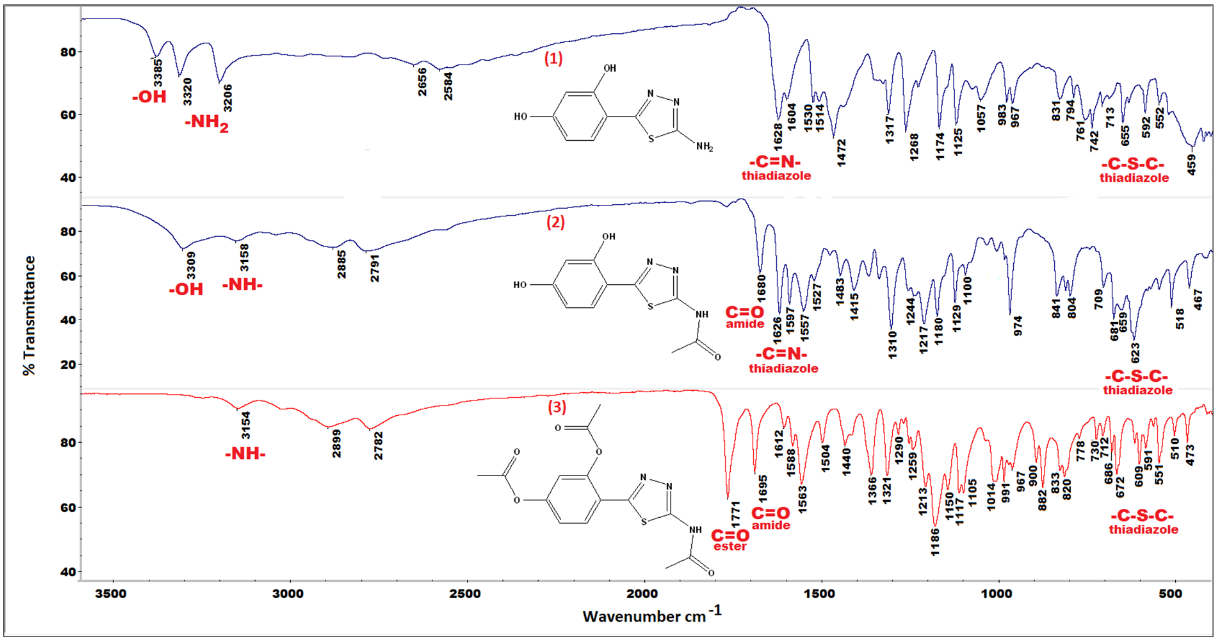

Figure S8. Comparison of the IR Spectra of thiadiazole derivatives **1**(top),**2**(middle),and **3** (bottom).

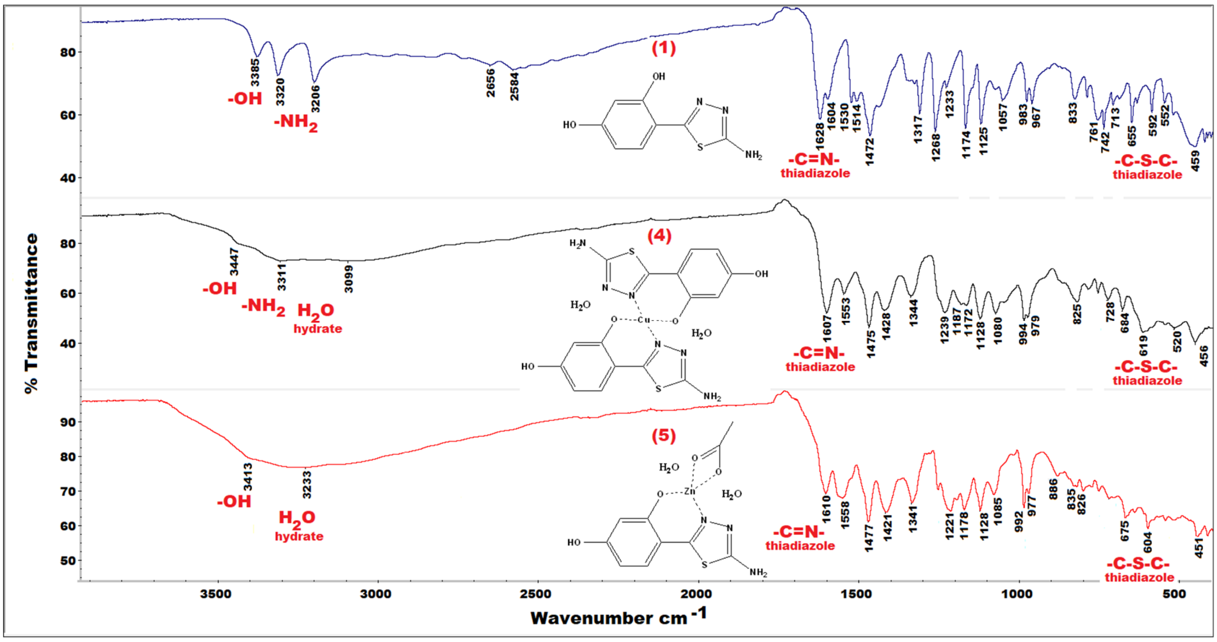

Figure 9. Comparison of the IR Spectra of thiadiazole **1**(top), and its corresponding Cu(II) and Zn (II) complexes **4** and **5** (middle and bottom, respectively).

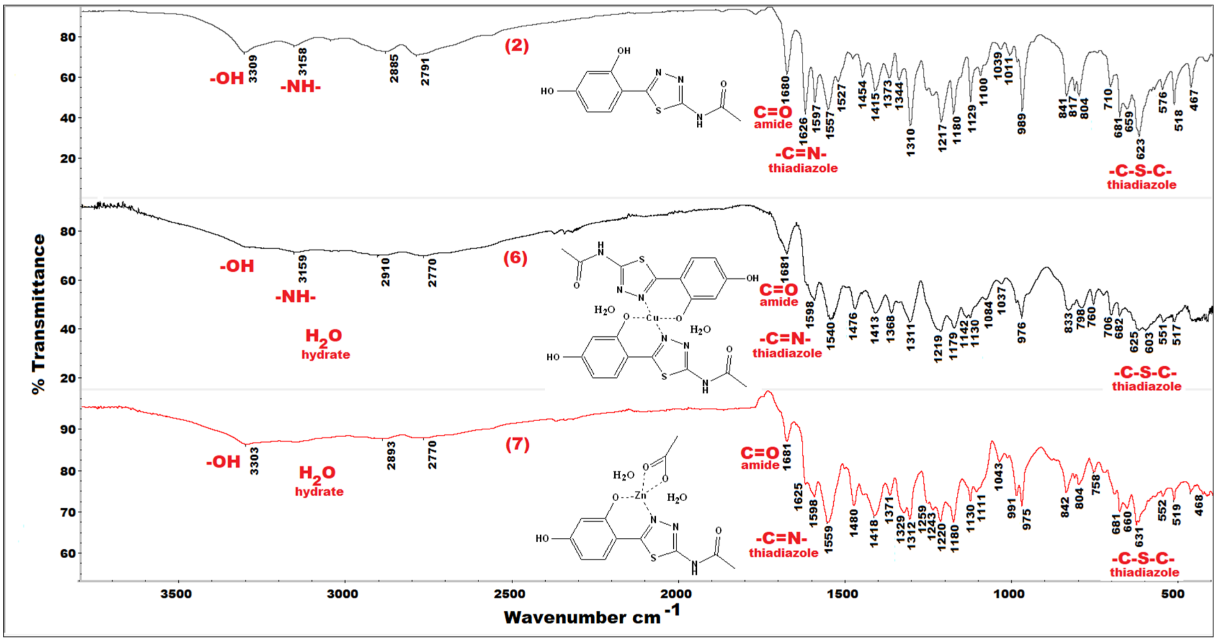

Figure 10. Comparison of the IR Spectra of thiadiazole **2** (top), and its corresponding Cu(II) and Zn (II) complexes **6** and **7** (middle and bottom, respectively).

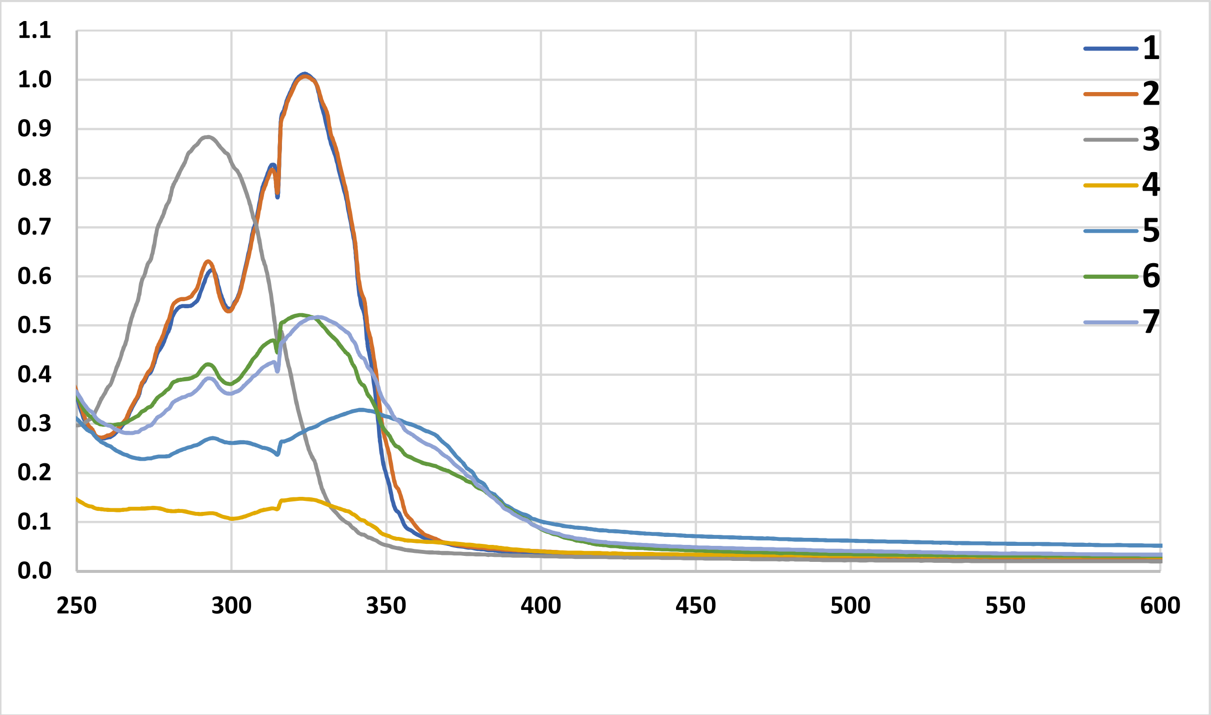

Figure 11. UV-Vis spectra of the thiadiazole derivatives **1**–**3** and their corresponding Cu(II) and Zn(II) complexes **4**–**7.**

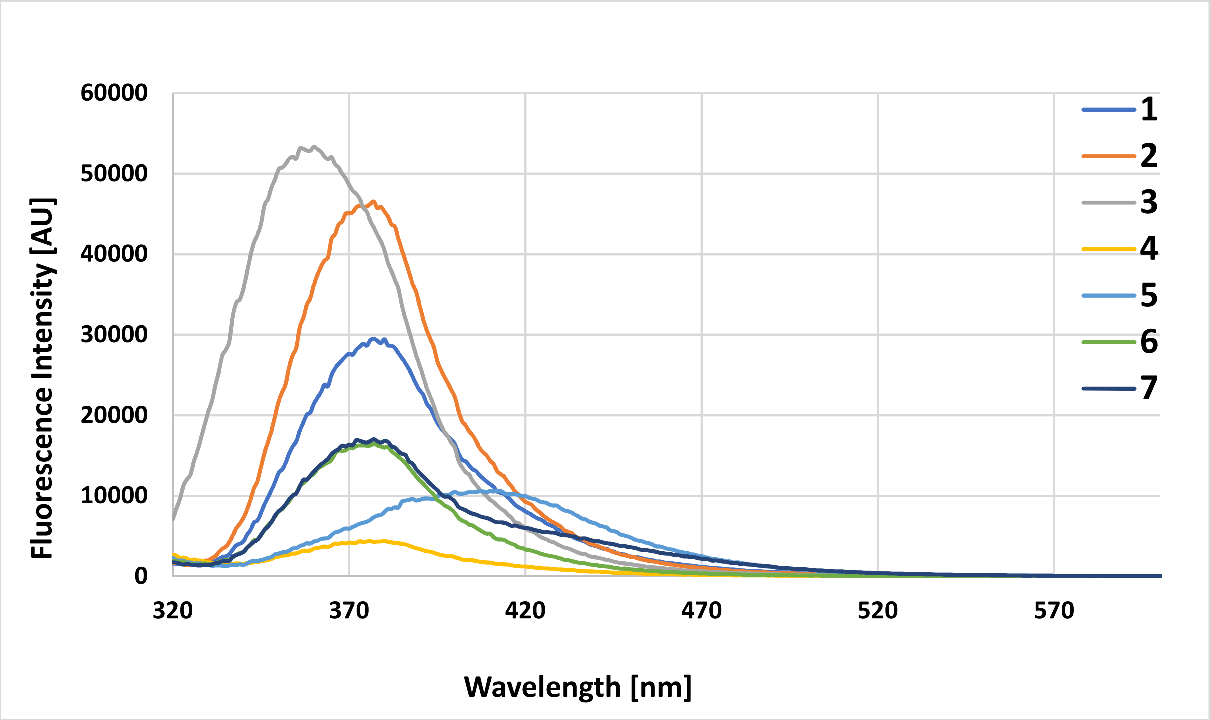

Figure 12. Steady state fluorescence spectra of the thiadiazole derivatives **1**–**3** and their corresponding Cu(II) and Zn(II) complexes **4**–**7**. The spectra recorded using the excitation wavelength λ_{Ex}= 290 nm.
